# Voting with Your Feet: The Impact of Urban Public Health Service Accessibility on the Permanent Migration Intentions of Rural Migrants in China

**DOI:** 10.3390/ijerph192214624

**Published:** 2022-11-08

**Authors:** Qingjun Zhao, Meijing Song, Hanrui Wang

**Affiliations:** 1 College of Economics and Management, Nanjing Agricultural University, Nanjing 210095, China; 2 College of Finance and Economics, Hainan Vocational University of Science and Technology, Haikou 571126, China

**Keywords:** public health service accessibility, permanent migration intention, rural migrants, China, voting with your feet

## Abstract

The accessibility of urban public health services is not only relevant to the health status of rural migrants but also plays an increasingly important role in their migration decisions. Most existing studies have focused on the effects of the level of public health service provision and parity on rural migrants’ migration behavior, ignoring the role of public health service accessibility. This paper systematically examines the overall impact, heterogeneous impact and mechanism of action of public health service accessibility on rural migrants’ intentions to migrate permanently based on data from the 2017 China Mobile Population Dynamics Monitoring Survey using probit, IVprobit, eprobit, omitted variable test model and KHB mediating effect model. It was found that: (1) public health service accessibility significantly increased rural migrants’ intentions to migrate permanently, and the results remained robust after using instrumental variables to mitigate endogeneity problems and omitted variable tests. (2) Heterogeneity analysis shows that public health service accessibility has a greater effect on enhancing the intentions to migrate permanently among females and rural migrants born in 1980 and later. (3) Further mechanism testing revealed that public health service accessibility could indirectly increase rural migrants’ intentions to migrate permanently by improving health habits, health status, identity, and social integration, with identity playing a greater indirect effect. The findings of this paper not only provide empirical evidence for the existence of Tiebout’s “voting with your feet” mechanism in China but also contribute to the scientific understanding of the role of equalization of public health services in the process of population migration.

## 1. Introduction

In China, hundreds of millions of rural migrants are constantly moving between urban and rural areas. How to make basic public health services reach the vast number of rural migrants is an extremely critical but weakest link in the construction of the current public health service system. It should be especially noted that internal migrants in China mainly refer to those whose household registration does not coincide with their place of residence, while rural migrants are internal migrants with agricultural household registration. According to the “Statistical Bulletin of the People’s Republic of China on National Economic and Social Development in 2021” issued by the National Bureau of Statistics of China, the total number of rural migrants in China will reach 292.51 million in 2021, accounting for 20.71% of the country’s total population. It has been found that the inadequate quantity and quality of public health service provision for rural migrants make the health status of rural migrants worrying [1,2,3,4]. Compared with urban residents, rural migrant groups are both more vulnerable to infectious diseases and more likely to become potential transmitters. Notably, unlike other countries, since 1958, China has implemented a household registration system that divides household attributes into agricultural and non-agricultural hukou based on geographic and family member relationships [5]. This institutional arrangement links social welfare benefits such as children’s education, employment services, social security, health care, and housing security to the household registration system. Therefore, the national basic public health service system is split into two relatively independent subsystems, urban and rural, and rural migrants can only obtain services and realize their rights from the rural basic health service system [6,7,8]. This means that rural migrants can only enjoy the same rights to public health services as local residents after achieving household registration migration. Therefore, in order to improve the accessibility of public health services for rural migrants, improve the health literacy of rural migrants and help this group enhance its competitiveness in integrating into cities, the government has formulated a series of public health service policies for the floating population, gradually providing equalized public health services to rural migrants. In 2013, the National Health and Family Planning Commission of China launched a pilot program for the equalization of basic public health services for the floating population in 40 cities across the country. In 2016, the Central Committee of the Communist Party of China and the State Council issued the “Healthy China 2030” Planning Outline, which emphasized the equalization of basic public health and family planning services for the floating population. In 2020, the “14th Five-Year Plan for National Economic and Social Development of the People’s Republic of China and the Outline of Vision 2035” raised a new level of building a strong public health system and improving national health promotion policies.

The majority of rural migrants leave their hometowns and enter the cities. In addition to obtaining better job opportunities and higher incomes, becoming urban residents is also one of the life goals that most of them strive for. The essence of the urbanization of rural migrants is to gradually allow the rural labor force to transfer to urban employment to enjoy the same basic public service rights as local citizens and to allow them to enjoy the full national treatment as citizens [9,10,11]. Tiebout’s “voting with feet” theory was the first to add local public services to the utility model of population migration, arguing that residents “buy” between different regions and choose the region where the combination of public goods and taxation best matches their preferences [12]. However, a large number of empirical literature shows that public health policies targeting rural migrants have not been fully implemented in places of inflow [13,14]. Rural migrants are more likely to suffer health threats due to high occupational risks and poor living environment and are likely to become blind spots for immunization, infectious disease prevention, and occupational health protection in the inflow areas [15,16]. The utilization effect of public health services for rural migrants can be regarded as the “touchstone” for testing the operation of the public health system. Whether the availability of public health services can help improve the intention of rural migrants to permanently migrate and to what extent they can promote the process of rural migrants’ citizenization can evaluate the quality of urban public health services to a certain extent. Unfortunately, current research focuses on evaluating the impact of public health services on rural migrants from the perspectives of the implementation progress, investment intensity, and level of equalization of public health services [17,18,19,20,21]. However, the accessibility of public health services, which is more important for rural migrants, and the role of accessibility of public health services in the permanent migration of rural migrants have been neglected. A comprehensive clarification of the impact of public health service accessibility in the inflow area on rural migrants’ intentions to migrate permanently and its logical chain not only can help analyze the paths and areas where public health service equalization policies produce welfare effects but also provide a reference for territorial governments to reasonably allocate public health service resources for the migrant population.

Therefore, we re-examine the mechanisms of the role of public health service parity in improving the survival of rural migrants and in facilitating the process of population migration. Specifically, we answer the following three questions: First, can public health service accessibility enhance rural migrants’ intentions to migrate permanently? Second, if there are certain positive effects, what are the possible mechanisms of action inherent in it? Third, are there heterogeneous characteristics of the positive effects of public health service accessibility within rural migrant groups? Compared with the existing research, our possible marginal contributions are: First, based on the perspective of the quality of public health services received by rural migrants, we systematically investigate the role of public health service accessibility in promoting rural migrants’ intentions to migrate permanently, which expands the research related to rural migrants’ intentions to migrate permanently. Secondly, based on the assessment of the basic effects, this paper deeply analyzes the mechanisms through which accessibility of public health services affects rural migrants’ intentions to migrate permanently from the perspectives of health literacy, health level, identity and social integration. At the same time, it also analyzes the heterogeneity of public health service accessibility in influencing rural migrants’ intentions to migrate permanently. The research in this paper can provide theoretical references and empirical evidence for the effective health promotion effects of public health policies for migrant populations.

The rest of this paper is structured as follows: The next section reviews the literature on access to public health services and rural migrants’ intention to migrate permanently and puts forward the research hypothesis. Section 3 introduces the primary variables, data sources, and estimation strategies. The empirical results are presented in Section 4. Section 5 discusses the main findings of this study and compares them with existing research. In last, Section 6 concludes the study with several policy implications.

## 2. Literature and Hypotheses

### 2.1. Literature Review

Regarding the influencing factors of rural migrant migration decision-making, scholars are mostly based on labor market segmentation theory [22], spatial location theory [23], push-pull theory [24], neoclassical economics theory [25], and new migration economics theory [26,27], selected the individual characteristics, flow characteristics, urban characteristics and macro-systems of rural migrants as explanatory variables for a comprehensive investigation. Existing studies have shown that the current state of the migrant population’s inability to settle in the inflowing cities is mainly the result of China’s unique household registration system barriers [28,29]. However, the household registration system is not the only factor that prevents rural migrants from settling in cities; mobility characteristics and urban characteristics also have important effects. Song et al. found an inverted U-shaped relationship between urban size and rural migrants’ intentions to settle [30]. By comparing the different migration decisions of laborers from three neighboring states in India when participating in the National Rural Employment Guarantee Program, Clémen et al. found that monetary mobility costs represented by fixed costs and living costs accounted for a large proportion of total migration costs. However, non-monetary mobility costs, such as being far away from relatives, accommodation, etc., seem to be more important for labor migration decisions [31]. Housing prices are also an important factor affecting population migration. Housing prices directly determine the cost of population migration. High housing prices will hinder the intentions of rural migrants to settle down [32,33]. With the increasingly serious air pollution problem and the continuous improvement of the public’s requirements for public environmental services, air quality has an increasing impact on the intentions of rural migrants to stay [34,35]. In addition, aspects such as gender, education level, family size, income level, and occupation also influence rural migrants’ migration decisions [36,37,38,39,40,41]. In recent years, with the continuous release of rural surplus labor, more and more attention has been paid to the urbanization of rural migrants flowing into cities, and public services have played an important role. Public services can have a significant impact on rural migrants’ intentions to settle. The higher the level of urban economic development and the better the basic public services, the stronger the settlement intentions of rural migrants [38,42]. The theory of “voting with your feet” in the Tiebout model first paid attention to the impact of local public service supply on population migration and believed that residents’ preference for public services chose the direction of migration and residence intentions through “voting with their feet” [12]. Research on tax capitalization has verified the existence of the theory of “voting with your feet” [43]. In fact, due to institutional constraints and insufficient capacity, rural migrants receive lower levels of public services than local urban residents [44]. The main reason for this is that China’s current system of basic public service provision is mostly tied to household registration, making it more difficult for rural migrants to move permanently. This creates a great obstacle for rural migrants and restricts the improvement of their welfare level. Only a few studies have empirically analyzed the impact of the equalization of public health services on the social integration of rural migrants, and they believe that the use of public health services can help improve the social integration of rural migrants [45].

Based on the literature, the research on the relationship between public services and rural migrant migration decision-making has been relatively abundant, but most of the existing studies focus on the overall impact of public services on population mobility. At the same time, these studies have combined multiple types of public services for exploration, and there are few relevant studies that focus specifically on public health services. Not only that, most of the explanatory variables in the existing research focus on the investment intensity and supply status of public health services, and there are relatively few analyses of their internal mechanisms, which are significantly different from the accessibility of public health services that we focus on. In China, rural migrant groups generally have low health literacy and relatively weak awareness of health care. They are “vulnerable” groups in health risk management and are more vulnerable to diseases. Therefore, the quality of public health services received by rural migrants should receive more attention.

### 2.2. Research Hypotheses

Population migration theory holds that income disparity is the main reason for population migration [23,25], and there is little difference in public services between regions and cities in developed countries, so public services do not have an important impact on population migration decisions. However, in developing countries such as China, there is an administrative center bias in the allocation of government resources, and public services vary greatly among cities. This difference in public services between cities has a more important impact on China’s population migration decision-making. However, China’s unique household registration system determines that citizens’ rights have territorial characteristics. The right of rural migrants to obtain public services such as medical services is based on the local household registration status [46,47]. It can be seen that the household registration system in China dictates that rural migrants need to move their household registration to the local area in order for them and their family members to enjoy the public services provided by the inflowing city. The core content of public health services is public health. Although most rural migrants have paid the medical insurance in their place of residence, the medical insurance in the place of residence has not played its due role for the rural migrants who work and live in other places for a long time, and the reimbursement in other places is extremely cumbersome and difficult to apply for. The right to health is an inclusive basic human right and the basic guarantee for human beings to live in dignity. Everyone has the right to enjoy the highest, fair, and most accessible standard of health. In China, public health services were originally restricted by the household registration system, serving only the local household population. In 2009, China’s national basic public health service project was launched, which began to equalize public health services for the migrant population and gradually provide social security and public health services to rural migrants. Previous studies have found that rural migrants who have obtained public services such as social security, community health records, health education, and medical security have stronger intentions to migrate [32]. Therefore, the improvement of the level of access to public health services in the inflow areas will help to enhance the “sense of acquisition” of rural migrants, thereby increasing their intentions to migrate permanently.

**Figure 1:**
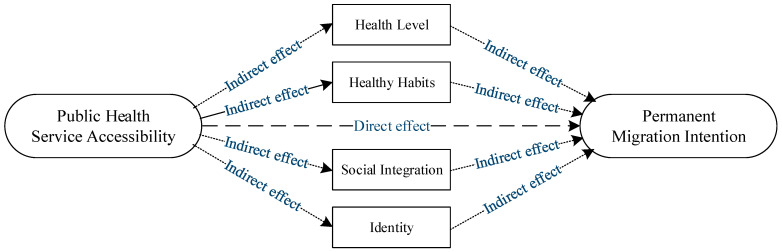
The logic of the impact of public health service accessibility on rural migrants’ permanent migration intention.

So, what is the mechanism by which access to public health services affects rural migrants’ intention to move permanently? Equalization policies for basic public health services that improve access to public health services for rural migrants need to be examined. On the one hand, the core content of public health services includes health education, health records and other specific content, and its main function or purpose is to safeguard the health rights and interests of rural migrants [13]. For rural migrants, providing public health education and establishing health records can enable rural migrants to obtain, understand and use health information more widely, thereby enriching their health knowledge, improving their health literacy, enabling them to develop good health habits and avoid health risks and ultimately improved health. At the same time, good health status can enrich the urban life of rural migrants, improve their quality of life and satisfaction, and enhance their ability to integrate into the economy. Therefore, the improvement of the accessibility of public health services in the inflow areas can help rural migrants acquire and absorb scientific and understandable medical and health knowledge, improve their health habits and improve their health level, and thus have a positive impact on the permanent migration intention of rural migrants. On the other hand, the equalization of basic public health services breaks through the shackles of the original household registration social power, enabling rural migrants to obtain basic public health services on an equal footing, narrowing the social rights gap with the local population, and maximizing their own welfare. In essence, the equalization of basic public health services is also a process of empowering rural migrants. It is a recognition of rural migrants’ social citizenship, which helps reduce the gap between rural migrants and locals, enhance their self-identity, and accelerate their social Fusion pace [20,45]. Therefore, improving the accessibility of public health services for rural migrants allows them to feel the care and assistance of the city where they live, shortens the psychological distance from the city, and enhances the identity and social integration of rural migrants, thereby increasing their intention to move permanently. According to the above analysis, this paper constructs the analysis framework shown in Figure 1 and proposes the following research hypotheses:

**Hypothesis 1**. *Access to public health services can significantly increase rural migrants’ intention to move permanently*.

**Hypothesis 2**. *Access to public health services increases rural migrants’ intention to move permanently by improving healthy habits, improving health, enhancing identity, and enhancing social integration*.

## 3. Materials and Methods

### 3.1. Data

The micro-data used in this article comes from the 2017 China Migrants Dynamic Survey (“CMDS”), a recent survey conducted by the National Health and Family Planning Commission of China, which is both authoritative and timely. The survey adopts the stratified, multi-stage, and scale-proportional PPS sampling method. The survey covers the inflow places where the floating population is relatively concentrated among the 31 provincial-level administrative units in mainland China. The data is collected from people aged 15 and above without household registration in the district (county, city). The survey content involves the basic information about the floating population and their family members, the scope and trend of migration, the utilization of essential public health services, and the management status of marriage, childbirth and family planning services, etc., which has both professional, scientific and large-scale characteristics, and can comprehensively describe the utilization of public health services of rural migrants in China. The total number of CMDS 2017 data samples is 169,989. Since this paper focuses on rural migrants who are working and doing business in urban areas, and only migrants who have lived locally for more than 6 months can enjoy basic public health services. Therefore, after excluding the samples with missing key variables, a total of 92,002 valid samples were screened.

### 3.2. Variables

The explained variable is permanent migration intention(“PMI”). The so-called permanent migration intention generally refers to the intention of rural migrants to settle down in their destination for a long time. In China, due to the restrictions of the household registration system, rural migrants can only enjoy the same rights as local residents if they obtain a local household registration. Therefore, rural migrants need to settle locally in order to gain institutional legitimacy for permanent migration. Drawing on the practice of existing literature [48,49], we will regard rural migrants who are willing to move their hukou to the inflow area or who are willing to settle in the inflow area as a permanent migration intention. Measured by the items in the CMDS 2017 questionnaire “If you meet the local settlement conditions, are you willing to move your household registration to the local area?” and “Do you plan to settle in the local area?” We consider their willingness to settle in their destination or to move hukou to the destination as a permanent migration intention, it is assigned a value of 1, and other cases are assigned a value of 0. Within the sample, 41,138 rural migrants have permanent migration intentions, accounting for 44.71%.

The explanatory variable is the accessibility of urban public health services(“PHSA”). This paper uses the “documentation of health records” to measure the accessibility of urban public health services for rural migrants. For a number of public health service projects, such as maternal and child health care management, the establishment of health records directly affects the type and content of health services enjoyed by rural migrants. In the questionnaire, this indicator was operationalized as “whether a resident health file has been established for you locally,” and the respondents answered, “yes, it has been established,” “not established, never heard of it,” “not established, but heard of it,” or “unclear.” This paper assigned “yes, has been established” a score of 1, and other cases are assigned 0. Within the sample, there were 25,476 rural migrants with health records, accounting for 27.69%.

Considering the possible endogeneity problem, this paper uses the instrumental variable method to test. The accessibility of public health services for rural migrants in the inflow areas mainly depends on the policy efforts of the local governments to provide public health services for the floating population. In 2013, the National Health and Family Planning Commission of China issued the Pilot Work Plan for the Equalization of Basic Public Services for Health and Family Planning for the Floating Population. Currently, there are 31 provincial-level administrative units in 44 cities with a high concentration of floating population in mainland China. Equalization of basic public services for population health and family planning (as shown in Table A2). Rural migrants in the above-mentioned 44 key cities can enjoy the same rights to public health services as local residents, which will greatly improve the accessibility and level of public health services for rural migrants. However, whether or not the inflow areas of rural migrants are selected as key cities generally does not directly affect the intention of rural migrants to move permanently. Therefore, we select “key cities for equalization of public health services” as an instrumental variable for the accessibility of public health services for rural migrants.

This paper considers health habits, health level, identity and social integration as the mechanisms by which access to public health services affects rural migrants’ intention to migrate permanently. Among them, healthy habits are measured by the questionnaire item “My hygiene habits are no different from local citizens,” and the option agrees with 1 and disagrees with 0. The health level is measured by the questionnaire item “How is your health status.” The options include “can’t take care of yourself,” “unhealthy, but can take care of yourself,” “basically healthy,” and “healthy,” which are assigned as 1, 2, 3, and 4 in turn. Identity identification was measured with the questionnaire item “I think I’m already a local,” assigning the option to agree as 1 and disagree as 0. Social integration was measured by the questionnaire item “I am very willing to integrate into the local area,” and the option was assigned a value of 1 for agreeing and 0 for disagreeing.

This paper controls for various potential confounding factors that may affect both the accessibility of public health services and the intention to permanently relocate among rural migrants, mainly including gender, age, marital status, education level, the scope of mobility, length of local residence, income level, medical care basic personal characteristics such as insurance, employment status and family size. In addition, the “China Urban Statistical Yearbook 2017” was matched with the CMDS 2017 data, and urban characteristics such as per capita GDP and urban population size were selected as control variables. The meaning of each variable and its descriptive statistics are shown in Table A1.

### 3.3. Empirical Strategies

#### 3.3.1. Main Effects Model

Since the explained variable intention to permanently migrate is a binary variable, the Probit model is used for estimation. The expression for this model is:

(1) $$y_{i}^{*}=a_{0}+a_{1}x_{i}+a_{2}s_{i}+\varepsilon_{i}$$

(2) $$y_{i}=\{\begin{array}{c} \;1,\;y_{i}^{*}>0\\ \;0,\;y_{i}^{*}\leq 0 \end{array}$$

As shown in Equation (1), $y_{i}^{*}\;$ represents the latent variable of the permanent migration intention of rural migrants. When $y_{i}^{*}$ > 0, $y_{i}^{*}$ = 1, otherwise $y_{i}^{*}$ = 0. $x_{i}$ represents the availability of public health services, $s_{i}$ is a series of control variables, including gender, age, marital status, education level, range of mobility, local residence time, income level, medical insurance, employment status, family size, per capita GDP and urban population size. $a_{j}\left(j=0,\;1,\;2\right)$ is the parameter to be estimated, and $\varepsilon_{i}$ is the random disturbance term.

#### 3.3.2. Instrument Variable Method

Of course, there may also be potential endogeneity in the above benchmark regressions. Specifically: First, the level of public health services enjoyed by rural migrants depends not only on the public service supply of the inflowing governments but also on the actual needs of rural migrants. However, we cannot exclude such a segment of rural migrants who are more motivated to access public health services because they have a strong desire to move permanently, a situation that would lead to reverse causation problems. Second, the factors affecting rural migrants’ decision on permanent migration intentions are more complex and difficult to control completely in the model. There may be other unobservable factors that can affect both rural migrants’ public health service accessibility and permanent migration intentions, resulting in the problem of omitted variables. Therefore, in order to reduce the inconsistency of parameter estimation and further improve the reliability of the estimation results, this paper attempts to use the IVprobit model to carry out regression analysis. The specific model settings are as follows:

(3) $$y_{i}^{*}=b_{0}+b_{1}x_{i}+b_{2}s_{i}+u_{i}$$

(4) $$x_{i}=c_{0}+c_{1}z_{i}+c_{2}s_{i}+v_{i}$$

(5) $$y_{i}=\{\begin{array}{c} \;1,\;y_{i}^{*}>0\\ \;0,\;y_{i}^{*}\leq 0 \end{array}$$

In Equations (3)–(5), $y_{i}$ is an observable dummy variable, $y_{i}^{*}$ is an unobservable latent variable, $x_{i}$ is the only endogenous variable in the model, $z_{i}$ is an instrumental variable, and $u_{i}$ and $v_{i}$ are random disturbances items.

At the same time, this paper also uses the Extended Probit (“Eprobit”) model in the Extended Regression Mode framework for re-estimation to test the robustness of the estimated results. The biggest advantage of the Eprobit model is that it can deal with the endogeneity of explanatory variables or control variables, the non-random distribution of policy variables in effect, and the endogenous sample selection problem. For a detailed introduction to the model, please refer to the regression tool “Eprobit” used in STATA.

In addition, we also solve the endogeneity problem caused by missing variables through another way of thinking: if there are missing variables, how big can the coefficient bias be? Will it affect the conclusion of this article? To this end, this paper uses the method proposed by Oster to test the potential missing variables and their impact on the regression results [50]; that is, when there are some unobservable missing variables in the regression model, it can be obtained by calculating the estimator $\beta^{*}$ approximately for consistent estimates of access to public health services on rural migrants’ intention to move permanently:

(6) $$\beta^{*}\approx \tilde{\beta }-\delta \left(\beta^{0}-\tilde{\beta }\right)\times \left(R_{max}-\tilde{R}\right)/\left(\tilde{R}-R^{0}\right)$$

Among them, $\beta^{*}$ represents the impact of the accessibility of public health services on the intention of rural migrants to permanently migrate, and $\beta^{0}$ and $R^{0}$ represent the parameter estimates and fittings of goodness when constrained control variables are added. $\tilde{\beta }$ and $\tilde{R}$ represent the parameter estimates and goodness of fit of public health service accessibility when all observable variables are added as control variables. δ represents the ratio of observable variables and unobservable variables to the explanatory power of rural migrants’ intention to move permanently. $R_{\max}$ represents the maximum goodness of fit of the regression equation when all omitted variables can be included in the model.

Following Oster’s suggestion [50], we adopt two identification strategies to test the effect of omitted variables: first, assume that $R_{\max}$ is 1.3 times, 1.4 times, 1.5 times and 1.6 times the goodness of fit of the current regression equation, and β = 0. If the value of δ is greater than 1, it means that the omitted variable will not change the influence of the explanatory variable on the explained variable. Second, it is also assumed that $R_{\max}$ is 1.3 times, 1.4 times, 1.5 times and 1.6 times the goodness of fit of the current regression equation, but when  δ = 1, if $\beta^{*}$ is within the 95% confidence interval of the estimated parameters, then the coefficient estimation showed that the results passed the robustness test.

#### 3.3.3. Mediation Effect Model

After analyzing the impact of public health service accessibility on the intention of rural migrants to migrate permanently, this paper further adopts Baron and Kenny’s mediation effect model [51], which uses health habits, health level, identity, and social integration as mediating variables. To investigate the specific paths through which healthy habits, health level, identity, and social integration affect rural migrants’ intention to permanently migrate, in addition to Equations (1) and (2), the following econometric models should be constructed:

(7) $$m_{i}=d_{0}+d_{1}x_{i}+d_{2}s_{i}+\varepsilon_{i}$$

(8) $$y_{i}^{*}=e_{0}+e_{1}x_{i}+e_{2}m_{i}+e_{3}s_{i}+\varepsilon_{i}$$

(9) $$y_{i}=\{\begin{array}{c} \;1,\;y_{i}^{*}>0\\ \;0,\;y_{i}^{*}\leq 0 \end{array}$$

Among them, $y_{i}^{*}$ represents the latent variable of the explained variable, when $y_{i}^{*}>0$, $y_{i}=1$, otherwise $y_{i}=0$. $x_{i}$ is the explanatory variable, and $m_{i}$ is the mediator variable, including health habits, health level, identity, and social integration. Since Equations (1) and (2) have confirmed that the accessibility of public health services significantly improves the intention of rural migrants to permanently migrate, if both $d_{1}$ and $e_{2}$ are significant, it indicates that there is an indirect effect. At this time, when $e_{2}$ is not significant, there is a complete mediation effect. When $e_{2}$ is significant, there is a partial mediation effect.

In addition, considering the mediation effect model proposed by Baron and Kenny [51], which is mainly aimed at the case where the explained variable is a continuous variable, in order to avoid potential bias in the estimation results, this paper further adopts the KHB method for the discrete variable case to test the robustness of the mediating effect [52].

## 4. Empirical Analysis

### 4.1. Baseline Regression Results

Table 1 reports the baseline regression results of the impact of public health service accessibility on rural migrants’ intention to migrate permanently. To verify the robustness of the regression results, this paper adopts a stepwise regression method. Column (1) only controls the core explanatory variables, column (2) includes individual characteristics, column (3) includes city characteristics, column (4) includes individual characteristics and city characteristics, column (5) is the marginal effect of the estimated outcome of column (4). From the results in Table 1, it can be seen that whether only the core explanatory variables are controlled, individual characteristics and urban characteristics are added, the impact of health records on the intention of rural migrants to permanently migrate is significantly positive at the level of 1%, indicating that the estimation results are robust. From the results in column (5), it can be seen that compared with rural migrants without health records, establishing health records will increase the intention of rural migrants to migrate permanently by 2.3%. The above results show that improving the accessibility of public health services can effectively increase the intention of rural migrants to move permanently. Hypothesis 1 is preliminarily confirmed.

Compared with rural female migrants, rural male migrants have a significantly lower intention to move permanently. Education level, local residence time, income level and medical insurance have a significant positive impact on the intention of rural migrants to migrate permanently. For migrants whose education is at the high school level or above, the longer the local residence time, the higher the income level, and for rural migrants with medical insurance, their intention to move permanently is significantly higher. The closer the range of movement, the lower the intention of rural migrants to move permanently. The results of urban characteristics show that the per capita GDP and urban population size of the inflow areas significantly increase the permanent migration intention of rural migrants.

### 4.2. Endogeneity

#### 4.2.1. Instrument Variable Result

Although in the benchmark regression analysis, the control variables that may have an impact on public health service access and permanent migration intention are controlled as much as possible, the empirical analysis may still face potential reverse causality and omitted variable bias in the process of empirical analysis. Therefore, we used the IVprobit model for further analysis. Table 2 reports the estimated results of the IVprobit model. From the estimation results of the first stage of the IVprobit model in column (1), the instrumental variables have a significant positive impact on health records, which means that the instrumental variables meet the correlation conditions. From the estimation results of the second stage of the IVprobit model in column (2), the endogeneity test parameters of health records are significant at the 1% level, indicating that health records are indeed endogenous variables, and endogenous variables are just identified. The Anderson–Rubin test statistic also shows that the model does not have the problem of weak instrumental variables, and the estimation results of the IVprobit model are more robust than the Probit model. From the estimated results, health records will significantly increase the intention of rural migrants to permanently migrate, and the estimated coefficient of the IVprobit model is 1.351, which is larger than the coefficient in column (4) of Table 1, and the standard error is also larger, indicating that the benchmark regression underestimated the effect of the accessibility of public health services on the intention of rural migrants to move permanently.

Since the endogenous variable health record is a binary variable, the use of the IVprobit model reduces the validity of the estimated results to a certain extent. To this end, this paper further uses the Eprobit model for estimation. From the regression results of endogenous variables of the Eprobit model in column (3), it can be seen that the instrumental variables have a significant positive impact on health records, which also verifies that the instrumental variables meet the correlation requirements. The main regression results in column (4) show that health records have a significant positive impact on the intention of rural migrants to permanently migrate, with a coefficient of 1.772, which is larger than the coefficient in column (2), which indicates that the IVprobit model does exist in the estimation Validity loss problem. The correlation test of residual items showed that the correlation between the regression model of endogenous variables and the main regression model was significant, indicating that health records were indeed endogenous variables. After endogenous treatment, health records had a significant positive effect on rural migrants’ intention to migrate permanently, a result that remains highly consistent with the IVprobit model and the estimates of the Probit model. It can be seen that the positive effect of public health service accessibility on the intention of rural migrants to migrate permanently is robust, and research hypothesis 1 has been further confirmed.

#### 4.2.2. Omitted Variables Checks

Since Oster’s method is mainly applicable to the case where the explanatory variable is a continuous variable [50], we treat the intention to permanently migrate as a continuous variable and use OLS regression. Table 3 shows that regardless of whether $R_{max}$ is 1.3 times, 1.4 times, 1.5 times, or 1.6 times, $\beta^{*}$ is within the 95% confidence interval of the estimated participation, and when β = 0, all δ are greater than 1, indicating that health records The coefficients of variables affecting the intention of rural migrants to move permanently are relatively stable. Therefore, it can be considered that even if there are missing variables, the judgment of this paper on the relationship between public health service accessibility and permanent migration intention is still robust; that is, public health service accessibility can significantly improve the permanent migration intention of rural migrants.

### 4.3. Robustness

In order to further verify the reliability of the empirical results, we also conducted robustness tests by adjusting the explanatory variables and the explained variables. The results are shown in Table 4. First, we conduct robustness tests using two variables that are highly correlated with the explanatory variables. The first is to measure the accessibility of public health services through the questionnaire item “Have you received health education on disease prevention and control in your current village/residential in the past year? (Health education specifically involves nine aspects, including occupational disease prevention, STD/AIDS prevention, reproductive health and contraception, tuberculosis prevention, smoking control, mental health, chronic disease prevention, maternal and child health care/prenatal care, and self-help in public emergencies)” The value of having received any health education is 0. The second is through the questionnaire item “Have you received follow-up for hypertension or type Ⅱ diabetes provided free of charge by the local community health service center (station)/township health center in the past year?” to measure access to public health services, assigning the option “received” a value of 1, and “not received” a value of 0. The results of the regression after adjusting for explanatory variables are shown in columns (1) and (2), and the results show that the conclusions in Table 1 are still reached even if different measures of access to public health services are replaced. In addition, we also adjusted the assignment method of the explained variables. First, the intention to settle down in the inflow area was assigned as 1, and the lack of intention or unwillingness to settle down = 0. The regression results are shown in column (3). The second is to assign the value of willingness to settle in the inflow area as 1, unwillingness or unwillingness to settle = 0, and the result of re-regression is shown in column (4). The estimated results show that health records are still very significant, and the coefficient is significantly positive, indicating that the accessibility of public health services still significantly improves the willingness of rural migrants to migrate permanently, which further confirms the robustness of the core conclusion.

### 4.4. Heterogeneous Effects

**Figure 2:**
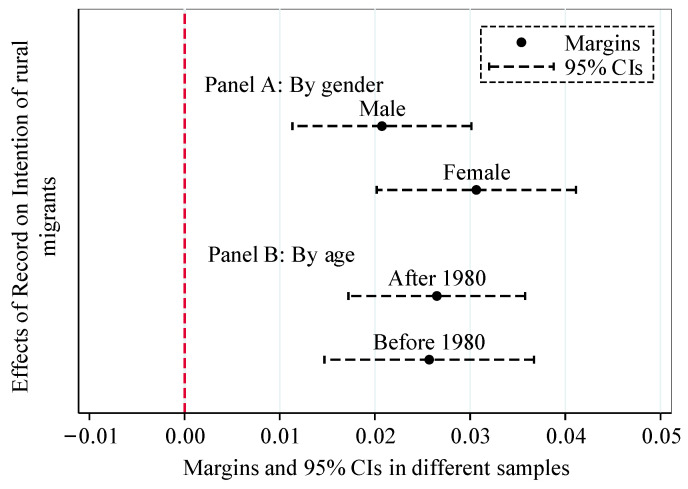
The heterogeneous effect of PHSA on PMI. (Note: The control variables introduced in the model fit were consistent with those in Table 1.)

The previous analysis has concluded that the accessibility of public health services helps to increase the intention of rural migrants to move permanently. However, it is worth noting that this is the average effect at the whole sample level, and the heterogeneity of the effect is not considered. In order to obtain more detailed research conclusions, we will group by gender and age and conduct a heterogeneity analysis. The specific estimation results are shown in Figure 2.

Panel A and Panel B showed the grouped regression results based on gender and age. The results show that access to public health services has a significant positive impact on the permanent migration intention of rural migrants of different genders and ages. However, access to public health services had a greater effect on permanent migration intentions for women born in or after 1980 than for men and rural migrants born before 1980. In addition, considering that there may be bias in examining heterogeneity by using the group regression method, we tested the difference between groups based on the test method of a seemingly uncorrelated model (“Suest”; as shown in Table A3). The Suest test shows that after grouping by gender and age, the coefficient differences between the groups are all significant at the 1% level, which further indicates that the impact of public health service accessibility on the permanent migration intention of rural migrants is different in different gender and age group.

### 4.5. Analysis of Mediating Effect

To test Hypothesis 2, we try to explore the possible mechanisms by which the accessibility of public health services affects rural migrants’ intention to permanently migrate from four aspects: health habits, health level, identity, and social integration.

As shown in Table 5, according to the test order of the mediation effect model, columns (1), (3), (5), and (7) respectively test the impact of health records on health habits, health level, identity and social integration, columns (2), (4), (6), and (8) test the effect of health records and four intermediary variables included in the model on the permanent migration intention of rural migrants. The results show that health records have a significant positive impact on the health habits of rural migrants, which means that the accessibility of public health services helps rural migrants develop healthy habits. Similarly, health records also have a significant positive impact on health, identity, and social integration, indicating that access to public health services can help rural migrants improve their health, identity, and social integration. The above results show that health habits, health level, identity, and social integration are all channels through which public health service accessibility affects rural migrants’ permanent migration intention.

In addition, we also decomposed the mediating effects of healthy habits, fitness level, identity and social integration based on the KHB method. As shown in Table 5, the estimated results of the KHB method are highly consistent with the estimated results obtained by the Baron and Kenny methods. The indirect effects of health level, identity, and social integration are all significant at the 1% level, and the coefficient sign is positive. Further analysis shows that the indirect effects of the four mediating variables, health habits, health level, identity and social integration, account for 5.97%, 1.49%,24.66% and 42.03% of the total effect of public health service accessibility on the permanent migration intention of rural migrants. It can be seen that the availability of public health services will not only directly increase the intention of rural migrants to permanently migrate but also improve the health habits of rural migrants, improve the health level of rural migrants, enhance the identity of rural migrants, and enhance the social status of rural migrants, indirectly increasing their intention to migrate permanently. This further confirms the robustness of the results of the mediation effect, and hypothesis 2 of this paper is confirmed.

## 5. Discussion

Providing urban public health services for rural migrants is an important path for the government to safeguard the health rights of rural migrants. To accomplish this goal, the “Healthy China 2030” plan emphasizes the equalization of basic public health and family planning services for the rural population, and the long-term rural population who have lived for six months or more is fully included in public health service projects and enjoys public health services on an equal footing. Most of the existing studies focused on the effects of public services such as public education, employment services, housing security, social services, and pension insurance on rural migrants’ permanent migration intentions [28,39], ignoring the role of public health services and the importance of public health services needs to be increased. Therefore, from the novel perspective of public health service accessibility, we systematically examined the overall effect, heterogeneous effect, and mechanism of action of public health service accessibility on rural migrants’ permanent migration intentions using the instrumental variables method, omitted variables test, and mediating effects model, and finally obtained robust and credible findings. Our work not only answers the key question of whether measures to equalize public health services for rural migrants actually work but also provides a factual basis for further equalization of public health services for rural migrants and empirical evidence for the existence of Tiebout’s “voting with feet” mechanism in China. Based on the CMDS 2017 data, this paper evaluates the impact and mechanism of the accessibility of public health services on the intention of rural migrants to relocate permanently. One of our most important findings is that increased access to urban public health services can significantly increase the probability of rural migrants’ intention to move permanently. After controlling possible endogeneity bias and passing a series of robustness tests, this boosting effect still exists. Public health service is one of the basic and core contents of the basic public services enjoyed by the people. It is closely related to the vital interests of the people. It can provide a platform for individual functional activities, is a means of developing feasible capabilities, and has important instrumental value. In China, rural migrant groups generally have low health literacy and relatively weak awareness of health care. They are “vulnerable” groups in health risk management and are more vulnerable to diseases. Therefore, the quality of public health services received by rural migrants should receive more attention. Our findings contribute not only to a deeper understanding of the development process of equalization of public health services for rural migrants but also to a scientific understanding of the role of equalization of public health services in promoting the migration of mobile populations.

In addition, we find that rural female migrants have a higher intention to move permanently, which is consistent with the majority of the literature, mainly because rural male migrants are more stressed than females to live in the city, and females are more likely to achieve social-type mobility by forming families in the city [53,54]. Rural migrants with higher education levels have relatively higher employment levels and income levels in cities and towns, have relatively higher expected economic benefits from long-term residence and are more willing to move permanently [55]. However, there is a certain cumulative effect of the residence time in the inflow area, which will make the rural migrants’ intention to stay transformed, that is, from long-term residence to permanent migration [56]. There are significant differences in the accessibility of medical security services between migrants and local residents. Citizenship and legal status have an important impact on their access to medical insurance. Therefore, medical insurance has an important impact on the social integration of migrants [57]. The larger the mobility of rural migrants and the higher their income levels, the stronger their ability to integrate into the economy and their intention to move permanently [58]. The higher the level of development and the larger the scale of the city, the more employment opportunities, higher income levels and better life services rural migrants have, and the more attractive they are for permanent migration [30,59].

This paper also finds that health habits, health level, identity and social integration are the mechanisms by which the accessibility of public health services affects the intention of rural migrants to migrate permanently. On the one hand, the improved accessibility of urban public health services can help rural migrants improve their health habits and improve their own health, thereby enhancing rural migrants’ intention to move permanently. Relevant studies have shown that health is the basic content of rural migrants’ human capital, which determines their citizenization ability to a certain extent. Rural migrants with better health status have a higher intention to settle down [60]. Compared with physical health, mental health has a greater impact on rural migrants’ short-term settlement intention. However, from the perspective of long-term settlement intention, the impact of physical health status is significantly more important. Our research shows that the accessibility of urban public health services significantly improves the health literacy of rural migrants, enabling them to develop good health habits, avoid health risks, and ultimately improve their health. On the other hand, the provision of equalized public health services to rural migrants is actually overcoming the limitations of the household registration system and giving rural migrants a type of social rights [61]. The city’s recognition of the local social citizenship of rural migrants, to a certain extent, helps to narrow the social distance between the urban and local population and rural migrants. Existing studies have also found that improving the openness of urban public services can help accelerate the social integration of migrants and local residents [62]. Enjoying high-quality public services can help improve the living welfare of migrants and promote better integration of migrants into urban society [45]. Access to good public services also enhances the social integration of migrants by reducing the cost of living in cities for workers. The research of this paper shows that the accessibility of public health services is an important manifestation of the equalization of public services, which highlights the recognition of the social citizenship of rural migrants in cities, and enhances the identity and social integration of rural migrants, so that rural migrants can become new citizens. And the identity of the owner is integrated into urban life, which significantly improves its intention to relocate permanently.

Finally, the heterogeneity results show that there is a certain gender and intergenerational heterogeneity in the impact of public health service access on rural migrants’ intention to migrate permanently. The improvement of the intention to migrate is even greater. This is mainly because, with the development of the family-oriented migration of China’s migrant population, more and more women are beginning to join the ranks of the migrant population. The decision-making power of rural female migrants has also increased, which is influencing the permanent migration decisions of their families [39]. In addition, women’s greater exposure to public health services compared to men′s will have a greater impact on their intentions to move permanently. At the same time, for rural migrants born before 1980, working in the city is often based on survival rationality, and the goal is to support their families. Although they live in the city, their hearts are in the countryside [63]. Compared with rural migrants born before 1980, rural migrants born in 1980 and later tend to yearn for urban life and integrate into cities [58]. Not only that, rural migrants born in and after 1980 have a stronger awareness of their rights and hope to receive equal treatment in cities [64]. Therefore, women and rural migrants born in 1980 and later are more sensitive to increased access to public health services which have a greater impact on their intention to move permanently.

## 6. Conclusions and Policy Implications

The core of China’s new urbanization is that rural migrants enter and settle in cities, enjoying the radiant light of basic public services. Existing studies all believe that providing public health services to rural migrants can help improve the level of citizenization of this group but ignore the accessibility of public health services, which to a certain extent, conceals the policy of equalization of basic public health services. Based on the CMDS 2017 data and from the perspective of health records, we systematically evaluated the impact of public health service accessibility on the intention of rural migrants to migrate permanently and analyzed the mechanism of action. We find that improved access to urban public health services increases rural migrants’ intention to settle permanently, and this effect is more pronounced among women and rural migrants born in 1980 and later. In addition, the impact of public health service accessibility on rural migrants’ intention to permanently migrate not only has a direct effect but also indirectly increases rural migrants’ intention to migrate permanently through healthy habits, health level, identity, and social integration.

The findings of this paper have important policy implications. Governments at all levels should start by improving the accessibility of public health services for rural migrants, continuously improving the public health service system, and gradually enabling rural migrants to enjoy urban public health services equally. First, the territorial government should strictly fulfill the responsibilities of equalization of public health services, with the improvement of the rural migrant health file information registration management system as an entry point, explore the establishment of a regional and inter-departmental rural migrant health information sharing mechanism, and strengthen the flow of related information between different regions. The exchange of basic public health services for the population will narrow the gap between regions, and establish a grid management system to facilitate accurate management in various regions, thereby improving service quality and efficiency. Second, it is necessary to improve the service attributes of public health-related institutions, strengthen the popularization of health education for rural migrants, and promote the social integration of rural migrants. Use multiple channels to further increase publicity efforts, improve the health awareness of the floating population, and increase the efficiency of public health services for rural migrants. At the same time, it actively guides rural migrants to participate in community activities, establishes the identity attributes of community owners, and strives to enhance their sense of urban belonging and identity.

## Figures and Tables

**Table 1:** Probit estimates of the effects of PHSA on PMI of rural migrants.

Variables	(1)	(2)	(3)	(4)	(5)
PHSA	0.029 ***	0.030 ***	0.078 ***	0.064 ***	0.023 ***
	(0.009)	(0.010)	(0.010)	(0.010)	(0.003)
Gender		−0.033 ***		−0.018 **	−0.006 **
		(0.009)		(0.009)	(0.003)
Age		−0.001		−0.001	−0.000
		(0.001)		(0.001)	(0.000)
Marriage		0.054 ***		0.017	0.006
		(0.015)		(0.015)	(0.005)
Education		0.183 ***		0.160 ***	0.057 ***
		(0.010)		(0.010)	(0.004)
Migration range		−0.139 ***		−0.029 ***	−0.010 ***
		(0.006)		(0.006)	(0.002)
Residence time		0.015 ***		0.014 ***	0.005 ***
		(0.001)		(0.001)	(0.000)
Income		0.073 ***		0.020 ***	0.007 ***
		(0.008)		(0.007)	(0.003)
Medicare		0.248 ***		0.165 ***	0.059 ***
		(0.011)		(0.011)	(0.004)
Occupation		−0.066 ***		−0.015	−0.005
		(0.009)		(0.010)	(0.003)
Family size		−0.002		−0.000	−0.000
		(0.005)		(0.005)	(0.002)
GDP			0.193 ***	0.152 ***	0.054 ***
			(0.011)	(0.011)	(0.004)
City size			0.242 ***	0.221 ***	0.078 ***
			(0.006)	(0.006)	(0.002)
Constant	−0.377 ***	−0.879 ***	−3.048 ***	−2.782 ***	
	(0.005)	(0.071)	(0.114)	(0.133)	
Pseudo R^2^	0.0001	0.0226	0.0380	0.0463	
Observations	92,002	92,002	92,002	92,002	92,002

Note: Robust standard errors in parentheses. *** *p* < 0.01, ** *p* < 0.05. Marginal effects are reported in column (5) of Table 1.

**Table 2:** The effects of PHSA on PMI: IV model test results.

Variables	IVprobit		Eprobit	
	(1)	(2)	(3)	(4)
PHSA		1.351 ***		1.772 ***
		(0.468)		(0.295)
Instrumental variable	0.022 ***		0.010 ***	
	(0.003)		(0.003)	
Wald test of exogeneity		9.000 ***		
AR weak Instrumental variable test		9.960 ***		
Corr (e. PHSA, e. PMI)				−0.774 ***
				(0.137)
Constant	0.308 ***	−3.160 ***	0.272 ***	−2.222 ***
	(0.048)	(0.170)	(0.002)	(0.409)
Control variables	YES	YES	YES	YES
Observations	92,002	92,002	92,002	92,002

Note: Robust standard errors in parentheses. *** *p* < 0.01.

**Table 3:** Omitted Variables Checks results.

Variables	Standard of Judgment	$R_{max}$				Pass The Test
		$1.3\tilde{R}$	$1.4\tilde{R}$	$1.5\tilde{R}$	$1.6\tilde{R}$	
PHSA	$\beta^{*}\left(R_{max},\delta \right)\in$ [0.0183, 0.0323]	0.0243	0.0240	0.0236	0.0233	YES
	δ > 1	21.6355	16.3251	13.1079	10.9499	YES

**Table 4:** The effects of PHSA on PMI: Robustness test results.

Variables	(1)	(2)	(3)	(4)
Health education	0.030 ***			
	(0.010)			
Health checkup		0.087 **		
		(0.035)		
PHSA			0.064 ***	0.086 ***
			(0.010)	(0.010)
Constant	−2.913 ***	−2.887 ***	−2.782 ***	−2.876 ***
	(0.132)	(0.131)	(0.133)	(0.149)
Control variables	YES	YES	YES	YES
Pseudo R^2^	0.0486	0.0486	0.0463	0.0730
Observations	92,002	92,002	92,002	92,002

Note: Robust standard errors in parentheses. ** *p* < 0.05, *** *p* < 0.01.

**Table 5:** The effects of PHSA on PMI: Mediating effect test results.

Variables	(1)	(2)	(3)	(4)	(5)	(6)	(7)	(8)
PHSA	0.119 ***	0.064 ***	0.101 ***	0.066 ***	0.213 ***	0.040 ***	0.128 ***	0.055 ***
	(0.011)	(0.010)	(0.011)	(0.010)	(0.011)	(0.010)	(0.015)	(0.010)
Healthy habits		0.123 ***						
		(0.011)						
Health level				0.029 ***				
				(0.010)				
Identity						0.459 ***		
						(0.010)		
Social Integration								1.085 ***
								(0.021)
Constant	−0.349 **	−2.963 ***		−3.009 ***	3.167 ***	−3.641 ***	0.807 ***	−3.917 ***
	(0.143)	(0.131)		(0.137)	(0.143)	(0.133)	(0.187)	(0.135)
Control variables	YES	YES	YES	YES	YES	YES	YES	YES
Pseudo R^2^	0.028	0.050	0.065	0.049	0.058	0.065	0.028	0.075
Total effect	0.067 ***		0.067 ***		0.069 ***		0.073 ***	
	(0.010)		(0.010)		(0.010)		(0.010)	
Direct effect	0.064 ***		0.066 ***		0.040 ***		0.055 ***	
	(0.010)		(0.010)		(0.010)		(0.010)	
Indirect effect	0.004 ***		0.001 ***		0.029 ***		0.018 ***	
	(0.000)		(0.000)		(0.002)		(0.002)	
Observations	92,002	92,002	92,002	92,002	92,002	92,002	92,002	92,002

Note: Robust standard errors in parentheses. ** *p* < 0.05, *** *p* < 0.01.

## Data Availability

Data sharing is not applicable to this article.

## Appendix A

**Table A1:** Descriptive statistics.

Variables	Value	Mean	Std.
PMI	Registered permanent residence or permanent settlement in the current city. 1 = Yes, 0 = No	0.447	0.497
PHSA	Is a resident health file established? 1 = Established, 0 = Other	0.277	0.447
Gender	1 = Male, 0 = Female	0.569	0.495
Age	Respondent’s age in 2017 (years)	36.033	9.828
Marriage	1 = Married, 0 = Unmarried	0.841	0.365
Education	1 = High school and above, 0 = Below high school	0.322	0.467
Migration range	1 = Inter-provincial mobility, 2 = Intra-provincial inter-city, 3 = Intra-city inter-county	1.666	0.752
Residence time	Duration of residence in the city (years)	6.214	5.924
Income	Logged family per capita income (Yuan)	7.594	0.693
Medicare	1 = Yes, 0 = No	0.203	0.402
Occupation	1 = Self-employed, 0 = Other	0.388	0.487
Family size	Number of family members living together	3.190	1.177
GDP	Logged GDP per capita in the current city of residence in 2016 (Yuan)	11.183	0.495
City size	The city size of the current city of residence. 1 =< 1 million, 2 = 1–5 million, 3 = 5–10 million, 4 => 10 million	2.016	0.928
Key cities	Key cities for equalization of basic public services for migrant population. 1 = Yes, 0 = No	0.437	0.496
Healthy habits	My personal hygiene habits are not different from those of local citizens. 1 = Yes, 0 = No	0.796	0.403
Health level	Self-reported health. 1 = Unable to take care of himself, 2 = Unhealthy, but life can be self, 3 = Basically healthy, 4 = Healthy	3.811	0.444
Identity	I am already a local resident. 1 = Yes, 0 = No	0.734	0.442
Social Integration	I am willing to integrate into this city. 1 = Yes, 0 = No	0.924	0.264

**Table A2:** List of key cities for the equalization of basic public health services for the floating population in China.

No.	Province	City	No.	Province	City
1	Beijing	Chaoyang	23	Fujian	Xiamen
2		Fengtai	24		Quanzhou
3	Tianjin	Binhai	25	Jiangxi	Nanchang
4		Jinan	26	Shandong	Qingdao
5	Hebei	Shijiazhuang	27	Henan	Zhengzhou
6	Shanxi	Taiyuan	28	Hubei	Wuhan
7	Inner Mongolia	Baotou	29	Hunan	Changsha
8	Liaoning	Dalian	30	Guangdong	Shenzhen
9	Jilin	Changchun	31		Zhongshan
10	Heilongjiang	Harbin	32	Guangxi	Guilin
11	Shanghai	Minhang	33	Hainan	Sanya
12		Yangpu	34	Chongqing	Yubei
13		Songjiang	35	Sichuan	Chengdu
14		Baoshan	36	Guizhou	Guiyang
15	Jiangsu	Nanjing	37	Yunnan	Yuxi
16		Suzhou	38	Tibet	Lhasa
17		Wuxi	39	Shaanxi	Xi ’an
18	Zhejiang	Hangzhou	40		Xianyang
19		Ningbo	41	Gansu	Lanzhou
20		Jiaxing	42	Qinghai	Xining
21		Shaoxing	43	Ningxia	Yinchuan
22	Anhui	Hefei	44	Xinjiang	Karamay

**Table A3:** Heterogeneous effects.

Variables	Male		Female		After 1980		Before 1980	
	Coef.	Margins	Coef.	Margins	Coef.	Margins	Coef.	Margins
	(1)	(2)	(3)	(4)	(5)	(6)	(7)	(8)
PHSA	0.055 ***	0.021 ***	0.083 ***	0.031 ***	0.071 ***	0.027 ***	0.069 ***	0.026 ***
	(0.013)	(0.005)	(0.014)	(0.005)	(0.013)	(0.005)	(0.015)	(0.006)
Constant	−2.807 ***		−3.108 ***		−3.560 ***		−2.182 ***	
	(0.172)		(0.204)		(0.182)		(0.191)	
Control variables	YES		YES		YES		YES	
Pseudo R^2^	0.046		0.054		0.083		0.026	
Suest test	120.550 ***				296.950 ***			
Observations	52,347	52,347	39,655	39,655	51,833	51,833	37,325	37,325

Note: Robut standard errors in parentheses. *** *p* < 0.01.
